# Abdominal Striae, Cesarean Scar, and Ultrasound Sliding Sign as Predictors of Uterine Adhesions in Women Undergoing Repeat Cesarean Section

**DOI:** 10.7759/cureus.98849

**Published:** 2025-12-09

**Authors:** Riya Kumari, Abirami V, Meena T S, Priyanka T

**Affiliations:** 1 Obstetrics and Gynaecology, Sree Balaji Medical College and Hospital, Chennai, IND; 2 Obstetrics and Gynaecology, Iswarya Fertility Centre, Chennai, IND

**Keywords:** complication of abdominal and pelvic surgeries, predictors of uterine adhesions, repeat caesarean section, scar characteristics, transabdominal adhesions in women, ultrasound sliding sign

## Abstract

Background

The escalating global cesarean section (CS) rate has led to a growing population undergoing repeat procedures, where postoperative adhesions are a major cause of surgical complications. Preoperative prediction of adhesions remains a significant challenge in obstetrics. This study aimed to evaluate the efficacy of non-invasive predictors - abdominal striae, previous CS scar characteristics, and the transabdominal ultrasound sliding sign - in forecasting intraoperative adhesions in women with a previous CS.

Methods

A prospective observational study was conducted among 155 pregnant women with at least one prior CS admitted for repeat cesarean delivery. Preoperatively, abdominal striae were graded using the Davey scoring system, and the previous CS scar was assessed using the Vancouver Scar Scale (VSS). A radiologist performed an ultrasound to determine the sliding sign. Intraoperative adhesion grading was performed using the Modified Tulandi and Lyell classification. Statistical analysis was done using Statistical Package for the Social Sciences (SPSS) version 23 (IBM Corp., Armonk, NY), with a p-value < 0.05 considered significant.

Results

Among the 155 participants, 68 (43.8%) had intra-abdominal adhesions, while 87 (56.2%) had none. The incidence of adhesions increased significantly with the number of previous CSs - 20 (26.7%) after one, 35 (51.5%) after two, and 13 (83.3%) after three or more CS (p = 0.007). A negative sliding sign was strongly associated with adhesions (60; 60.6%) compared to a positive sign (8; 14.3%, p < 0.001). Similarly, severe striae were linked to a higher adhesion rate (56; 62.9%, p < 0.001), and VSS >4 indicated significantly more adhesions (54; 58.1%, p < 0.001).

Conclusion

The study concludes that the preoperative assessment of abdominal striae, previous cesarean scar quality, and the ultrasound sliding sign provides a simple, non-invasive, and effective triad for predicting uterine adhesions. Integrating these markers into clinical practice can enhance preoperative risk stratification, optimize surgical planning, and improve patient safety during repeat CSs.

## Introduction

In recent decades, the global rate of cesarean section (CS) has increased dramatically, transforming from a life-saving procedure to one of the most commonly performed surgeries worldwide. According to data obtained from 150 countries, CS rates range widely from 7.3% to as high as 40.5%, reflecting considerable variation in obstetric practice and healthcare access across regions [1]. While cesarean delivery remains an essential intervention for preventing maternal and fetal morbidity in complicated pregnancies, the escalating trend has raised concerns regarding its long-term maternal consequences. One of the major contributors to the rising overall rate is the growing number of repeat CSs, as many women with a prior cesarean are often scheduled for subsequent surgical deliveries rather than attempting vaginal birth after cesarean (VBAC).

Among the multiple postoperative complications associated with abdominal and pelvic surgeries, adhesion formation is by far the most frequent and clinically significant [2,3]. Adhesions are fibrous bands that develop between tissues and organs following surgical trauma, leading to distorted anatomy, restricted mobility, and potential complications during subsequent operations. In obstetric practice, adhesions following CS can involve the anterior uterine wall, bladder, omentum, or bowel, thereby obscuring surgical planes and complicating subsequent surgeries. Literature suggests that adhesion formation after the first CS may occur in approximately 7% of cases, but this risk increases dramatically with each additional surgery, rising to 68% after the third CS [4].

The presence of dense or vascular adhesions during repeat cesarean delivery increases the risk of several intraoperative and postoperative complications, including injury to the bladder and bowel, increased blood loss, prolonged operative time, infection, and greater postoperative morbidity [5,6]. Furthermore, severe adhesions may necessitate intraoperative assistance from other surgical specialties, increase the likelihood of hysterectomy, and adversely affect perinatal outcomes. For the operating obstetrician, predicting the presence and severity of adhesions before surgery is crucial, as it allows appropriate surgical planning, resource allocation, and risk counselling for the patient. However, despite their high clinical relevance, there is currently no universally accepted or reliable preoperative method for predicting intra-abdominal adhesions in women with a history of previous CS.

Traditionally, the degree of adhesion formation can only be confirmed intraoperatively, leaving obstetricians unprepared for the potential surgical difficulties. Various indirect parameters, such as the number of prior CSs, time interval since the last surgery, and previous wound infections, have been explored, but their predictive accuracy remains poor [7]. In this context, identifying non-invasive and reproducible clinical or imaging markers that could anticipate the presence of adhesions is of great practical significance.

Recent literature has explored several potential predictive markers, including abdominal striae (stretch marks), the appearance of previous cesarean scars, and the ultrasound sliding sign [8,9]. Abdominal striae, caused by dermal stretching and collagen disruption during pregnancy, may reflect underlying connective tissue remodeling and have been postulated to correlate with the risk of adhesion formation. Similarly, the external appearance of the previous cesarean scar-whether flat, depressed, or raised-could indirectly indicate the extent of internal tissue healing or fibrosis.

Among imaging techniques, transabdominal ultrasound evaluation of the sliding sign has emerged as a simple, bedside, non-invasive method for assessing visceral adhesions. The sliding sign refers to the visible movement of the uterus against the abdominal wall during respiration; its absence or restriction suggests the presence of adhesions between the uterus and anterior abdominal wall. Several studies in gynecologic and infertility settings have validated the utility of this sign for predicting pelvic adhesions, but its application in obstetric populations, particularly in women undergoing repeat CS, remains underexplored [8-10].

Hence, combining these parameters, clinical indicators such as abdominal striae and scar morphology, together with a dynamic ultrasound marker like the sliding sign, may provide a feasible and accurate approach for anticipating adhesions before surgery. Such a composite evaluation would allow obstetricians to anticipate surgical challenges, ensure the availability of senior surgeons and multidisciplinary support, and counsel patients more effectively regarding intraoperative risks. Despite the potential of these indicators, current literature offers limited data correlating external abdominal features and ultrasound findings with intraoperative adhesion grades in pregnant women scheduled for repeat CS. Most studies available are either retrospective, involve small sample sizes, or focus on non-pregnant gynecologic patients [7-10]. Therefore, there exists a clear gap in evidence regarding the predictive reliability of these easily assessable clinical and imaging parameters in the obstetric population.

The rising global cesarean rate has inevitably led to a growing population of women at risk of adhesion-related complications. In low- and middle-income countries like India, where access to advanced imaging or preoperative laparoscopy is limited, a simple, cost-effective, and bedside method for predicting adhesions could significantly enhance maternal safety. The novelty of the present study lies in its integrated assessment of external abdominal signs (striae and scar characteristics) and ultrasonographic sliding sign as preoperative predictors of intra-abdominal adhesions. To our knowledge, limited studies have prospectively evaluated these combined predictors in a pregnant cohort undergoing repeat CS in the Indian population. This study aimed to evaluate the predictive role of abdominal striae, previous cesarean scar characteristics, and ultrasound sliding sign in identifying uterine and adjacent structure adhesions among pregnant women planned for repeat CS, and to determine the strength of association between these parameters and intraoperative findings.

## Materials and methods

Study design and setting

This was a hospital-based prospective observational study conducted in the Department of Obstetrics and Gynaecology at a tertiary care teaching hospital catering to both urban and semi-urban populations. The hospital serves as a referral center for obstetric and surgical emergencies, thus ensuring adequate representation of women undergoing repeat cesarean deliveries. The study aimed to investigate the association between external abdominal signs, namely abdominal striae and previous cesarean scar appearance, and ultrasonographic sliding sign, with intraoperative findings of uterine and adjacent structure adhesions. The study design was chosen to allow real-time assessment of preoperative and intraoperative parameters in the same participants.

Study duration and ethical considerations

The study was conducted over a six-month period, from October 2024 to March 2025. Ethical clearance was obtained from the Institutional Human Ethics Committee of Sree Balaji Medical College and Hospital (Ref: 002/SBMC/IHEC/2023/2521) prior to the initiation of the research. All participants were informed about the nature and purpose of the study, and written informed consent was obtained from each patient in their preferred language. Confidentiality was maintained throughout the research process, and all procedures conformed to the ethical standards of the Declaration of Helsinki.

Study population and sample size

The study population comprised pregnant women aged 20-40 years with a history of at least one prior CS who were admitted for elective repeat cesarean delivery in the second or third trimester. The sample size was determined using Dobson’s single-proportion formula, where Z = 1.96 for a 95% confidence interval, p = 0.68 (based on a prior study reporting adhesion rates after multiple cesareans [4], and d = 0.09 (absolute precision). The calculated sample size was 155 participants. Participants were selected using a consecutive sampling method to avoid selection bias and to ensure the inclusion of all eligible women during the study period.

Inclusion and exclusion criteria

The study included pregnant women aged 20 to 40 years with singleton pregnancies who had a history of at least one previous lower segment cesarean section (LSCS) and were scheduled for either elective or emergency repeat cesarean delivery. These participants were selected as they represented the target population at risk for postoperative adhesions and provided a uniform clinical context for evaluating preoperative predictors. Women were excluded if they had a history of prior pelvic or abdominal surgeries other than cesarean delivery, such as myomectomy or appendectomy, to avoid confounding adhesion sources. Similarly, patients with uterine anomalies, placenta accreta spectrum disorders, or ruptured uterus were excluded due to their potential to independently alter uterine or peritoneal anatomy. Those with severe preeclampsia, diabetes mellitus, or connective tissue disorders known to affect wound healing and skin integrity were also excluded to ensure the accuracy of external predictors like striae and scar morphology. Additionally, women with incomplete medical records or who declined participation were not included in the study.

Clinical evaluation and data collection

A structured, pre-validated proforma was used to collect sociodemographic, obstetric, and clinical details, which is attached in the Appendices. Data recorded included maternal age, gravidity, parity, gestational age, inter-pregnancy interval, and number of previous CSs. Particular attention was paid to body mass index (BMI) and the presence of comorbidities that may influence scar healing. Each participant underwent a thorough clinical abdominal examination. The abdominal striae were assessed visually and graded using the Davey scoring system, which divides the abdomen into quadrants and scores striae from 0 (none) to 2 (severe) in each quadrant, giving a maximum possible score of 8 [11]. Based on the total score, participants were categorized as having no striae, mild, moderate, or severe striae.

The previous cesarean scar was inspected for color, contour, and texture. Scar morphology was categorized as flat, raised/hypertrophic, or depressed/atrophic. Any irregular pigmentation or keloid formation was also noted, as these external scar features may reflect deeper fibrotic or adhesive processes.

Ultrasound evaluation of sliding sign

All participants underwent preoperative transabdominal ultrasonography using a 3.5-5 MHz curvilinear probe on a high-resolution ultrasound system (GE Voluson P8, Wheeling, IL). The sliding sign was assessed in the sagittal plane by placing the transducer just above the pubic symphysis with the patient in a semi-recumbent position. During quiet respiration, the movement of the uterus relative to the posterior aspect of the abdominal wall and bladder was observed. The sliding sign was recorded as positive, smooth sliding of the uterus against the abdominal wall during inspiration and expiration, and negative, restricted or absent sliding movement, suggesting adhesions between the uterus and anterior abdominal wall. Ultrasound examinations were performed independently by two radiologists, each with three to five years of experience in obstetric and gynecologic imaging, who were blinded to the patients’ clinical details. Interobserver discrepancies were resolved by consensus.

The Vancouver Scar Scale (VSS) was applied to assess the external appearance of the previous CS scar. Originally developed by Sullivan et al. for clinical evaluation of burn and surgical scars [12], the VSS objectively quantifies four scar characteristics: vascularity, pigmentation, pliability, and height/thickness. Each parameter is assigned a numerical score, and the total VSS ranges from 0 to 13, with higher scores indicating poorer scar quality or hypertrophic changes. In this study, a VSS score ≤4 was considered indicative of a normal or well-healed scar, while a score >4 suggested hypertrophic or fibrotic scar formation. Assessment was performed under good lighting conditions with the patient in a supine position, and the scoring was independently verified by two trained observers to ensure consistency. The VSS has been previously validated for use in obstetric populations and correlates with histological evidence of dermal fibrosis.

Intraoperative assessment of adhesions

During the CS, the intraoperative appearance and extent of adhesions were evaluated by the operating surgeon, who was blinded to the preoperative ultrasound and clinical findings. Adhesions were graded using the Modified Tulandi and Lyell classification system [13]: Grade 0: no adhesions; Grade 1: filmy, avascular adhesions easily separable by blunt dissection; Grade 2: dense, vascular adhesions requiring sharp dissection but limited to one area; and Grade 3: extensive, thick, vascular adhesions involving multiple structures or obscuring surgical planes.

The location of adhesions (between the uterus and bladder, omentum, or abdominal wall) and the time required for peritoneal entry were also documented.

Statistical analysis

Data analysis was performed using the Statistical Package for the Social Sciences (SPSS) version 26.0 (IBM Corp., Armonk, NY). Continuous variables, such as age and BMI, were expressed as mean ± standard deviation (SD), while categorical variables were expressed as frequencies and percentages. The Shapiro-Wilk test was applied to check normality. The Chi-square test (χ²) or Fisher’s exact test was used to compare categorical variables, such as the relationship between sliding sign and adhesion presence. A p-value < 0.05 was considered statistically significant.

## Results

The study included 155 pregnant women scheduled for repeat CS. The mean age of participants was 27.5 ± 4.8 years, with the majority in their late twenties. The mean BMI was 22.7 ± 3.5 kg/m², indicating that most women were within the normal weight range. The mean gestational age at examination was 37.2 ± 1.2 weeks, showing that most were assessed during term gestation. With respect to obstetric history, 75 women (48.4%) had undergone one previous CS, 68 women (43.9%) had two prior cesareans, and 12 women (7.7%) had three or more previous CSs. This distribution highlights that nearly 92% of participants had one or two prior cesarean deliveries, reflecting a typical obstetric profile of women presenting for repeat CS in tertiary care settings (Table 1).

**Table 1:** Demographic and Obstetric Characteristics of the Study Population (n = 155)

Variable	n (%)/mean ± SD
Age (years)	27.5 ± 4.8
Body Mass Index (kg/m²)	22.7 ± 3.5
Gestational Age at Examination (weeks)	37.2 ± 1.2
No. of Previous Cesarean Sections	
1	75 (48.4%)
2	68 (43.9%)
≥3	12 (7.7%)

Among the 155 participants, the sliding sign was positive in 56 women (36.1%) and negative in 99 women (63.9%), indicating that a majority exhibited restricted uterine mobility suggestive of adhesions. Based on Davey’s score for abdominal striae, 33 women (21.3%) showed no striae, 33 (21.3%) had mild striae, and 89 (57.4%) had severe striae. According to the VSS, 93 women (60.0%) had scores greater than 4, suggesting hypertrophic or raised scars, while 62 women (40.0%) had scores ≤4, indicating better scar healing (Table 2).

**Table 2:** Distribution of Sliding Sign, Davey’s Score, and Vancouver Scar Score Among Study Participants (n = 155)

Parameter	Category	n (%)
Sliding Sign	Positive	56 (36.1%)
	Negative	99 (63.9%)
Davey’s Score	None	33 (21.3%)
	Mild	33 (21.3%)
	Severe	89 (57.4%)
Vancouver Scar Score	≤4	62 (40.0%)
	>4	93 (60.0%)

**Figure 1:**
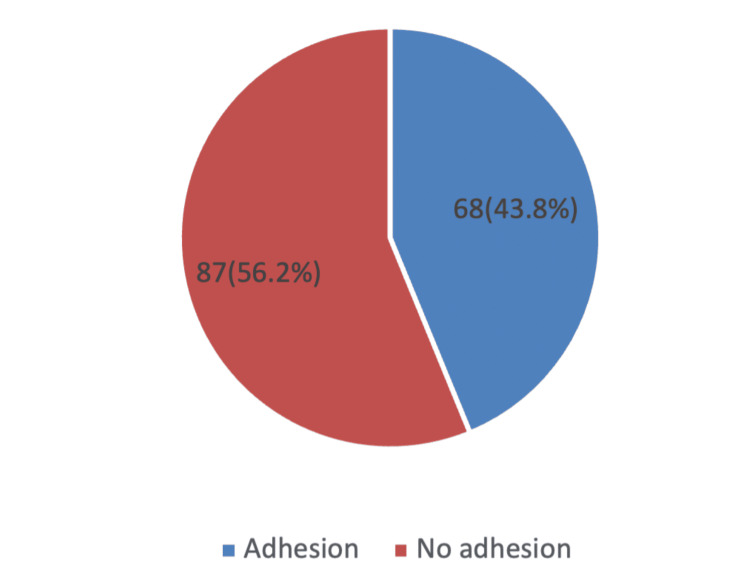
Distribution of Adhesions in Repeated Cesarean Section Among the Study Population (n = 155)

Figure 1 in the present study shows that intraoperative assessment revealed that adhesions were present in 68 women (43.8%), while 87 women (56.2%) showed no evidence of adhesions during repeat CS. This indicates that nearly two-fifths of the study population experienced varying degrees of adhesion formation following previous cesarean deliveries. 

Table 3 shows the association between adhesions and the parameters. Incidence of adhesions increased significantly with a higher number of previous CSs (χ² = 9.84, p = 0.007). Women with a negative sliding sign had a markedly higher adhesion rate (60, 60.6%) compared to those with a positive sliding sign (eight, 14.3%), showing strong statistical significance (p < 0.001). Similarly, severe Davey’s scores indicating extensive striae were associated with a greater likelihood of adhesions (62.9% vs. 18.2%, p < 0.001). A Vancouver Scar Score >4, reflecting hypertrophic or poorly healed scars, was also significantly correlated with intra-abdominal adhesions (58.1% vs. 22.6%, p < 0.001).

**Table 3:** Association Between Adhesions and Clinical/Imaging Parameters (n = 155) *p-value < 0.05 is statistically significant.

Variables	Categories	Adhesions present n (%)	No adhesions n (%)	χ² value	p-value
Number of Previous Cesarean Sections	1	20 (26.7%)	55 (73.3%)	9.84	0.007*
	2	35 (51.5%)	33 (48.5%)		
	≥3	13 (83.3%)	2 (16.7%)		
Sliding Sign	Positive	8 (14.3%)	48 (85.7%)	29.62	<0.001*
	Negative	60 (60.6%)	39 (39.4%)		
Davey’s Score (Striae Gravidarum)	None/Mild	12 (18.2%)	54 (81.8%)	24.11	<0.001*
	Severe	56 (62.9%)	33 (37.1%)		
Vancouver Scar Score	≤4	14 (22.6%)	48 (77.4%)	15.48	<0.001*
	>4	54 (58.1%)	39 (41.9%)		

## Discussion

The escalating global CS rate has precipitated a corresponding increase in the number of women presenting for repeat surgeries, making the management of associated complications, particularly postoperative adhesions, a critical aspect of modern obstetrics. Our prospective observational study sought to address this challenge by evaluating the efficacy of simple, non-invasive preoperative markers-abdominal striae, previous cesarean scar characteristics, and the ultrasound sliding sign-in predicting intra-abdominal adhesions in pregnant women with a history of prior cesarean delivery. The principal findings of our study demonstrate a statistically significant association between each of these predictors and the presence of intraoperative adhesions, thereby validating their potential utility in clinical risk stratification.

The demographic profile of our study population, with a mean age of 27.5 years and a normal mean BMI, is representative of a typical obstetric cohort in a tertiary care setting in India. The finding that 43.8% of our participants had intraoperative adhesions aligns with the existing literature, which reports a wide range of adhesion prevalence (7% to 68%) depending on the number of previous surgeries [4]. Our data reinforces the established correlation between the number of previous CSs and the risk of adhesion formation. We observed a dramatic increase in adhesion rates from 26.7% in women with one previous CS to 83.3% in those with three or more, a trend that is consistently documented. Tulandi and Lyell have postulated that each successive surgery incites a renewed inflammatory response and fibrotic reaction, leading to more extensive and denser adhesions [13]. This escalating risk underscores the imperative for careful preoperative planning in multigravid women.

The most potent predictor identified in our study was the transabdominal ultrasound "sliding sign." A negative sliding sign was present in 63.9% of our cohort and was strongly associated with adhesions, with 60.6% of women with a negative sign having confirmed intraoperative adhesions. Conversely, a positive sliding sign was a reliable indicator of the absence of significant adhesions, with a negative predictive value of 85.7%. This is in concordance with a study conducted by Alcázar et al., which described the "sliding sign" in the context of deep infiltrating endometriosis, finding it highly predictive of pouch of Douglas obliteration [14]. Our study successfully translates this concept to the obstetric population, demonstrating its utility in predicting anterior abdominal wall and bladder flap adhesions prior to repeat CS. The physiological basis for this is straightforward: adhesions between the uterine serosa and the posterior aspect of the abdominal wall or bladder tether the uterus, restricting its free movement against these structures during respiration. The high statistical significance (p < 0.001) of this association confirms that ultrasonographic assessment of visceral slide is a simple, rapid, and valuable bedside tool for anticipating surgical difficulty.

Our investigation also revealed a significant correlation between the severity of abdominal striae, as graded by the Davey scoring system, and the likelihood of adhesions. Women with severe striae had a 62.9% incidence of adhesions compared to only 18.2% in those with none or mild striae. This finding suggests a possible shared pathophysiology involving systemic connective tissue disposition. Abdominal striae gravidarum (stretch marks) result from dermal elastic fiber rupture and collagen reorganization under the influence of hormonal changes and mechanical stretching [15]. It is plausible that women with a genetic or constitutional predisposition to aberrant collagen remodeling and fibroblast activity may manifest this tendency both cutaneously (as striae) and intra-abdominally (as adhesions). This "common collagen hypothesis" is supported by studies linking striae to pelvic organ prolapse, another condition of connective tissue weakness [15,16]. Celik et al. similarly found that the presence and severity of striae were associated with a higher incidence of surgical difficulty in repeat CSs, although their study did not use a standardized scoring system [17]. Our use of the Davey score provides a more objective and reproducible method for this assessment, strengthening the evidence for this clinical sign.

Furthermore, the external appearance of the previous cesarean scar, quantified using the VSS, emerged as another significant predictor. A VSS score >4, indicative of hypertrophic or poor-quality scarring, was associated with a 58.1% adhesion rate, compared to 22.6% in women with well-healed (VSS ≤4) scars. This correlation implies that the biological processes governing dermal wound healing may mirror those occurring at the deeper fascial and peritoneal levels. Hypertrophic scarring is characterized by excessive deposition of collagen and other extracellular matrix components, driven by a prolonged inflammatory phase and dysregulated fibroblast function [18]. It is biologically consistent that individuals prone to this hyper-fibrotic response in the skin would exhibit a similar tendency to form fibrous adhesions intra-abdominally. Our findings are corroborated by a study by Abbas et al. [19], who reported that an avulsed or depressed scar was a predictor of bladder adhesions. The use of the VSS, a validated and multi-parameter tool, in our study adds a layer of objectivity and reproducibility that was lacking in earlier studies that relied on subjective scar descriptions.

The clinical implications of our findings are substantial. In resource-limited settings like India, where access to advanced imaging like MRI or diagnostic laparoscopy is constrained, the combination of a careful abdominal examination (for striae and scar) and a simple ultrasound assessment (for the sliding sign) provides a powerful, low-cost triad for preoperative risk assessment. Identifying a high-risk patient preoperatively allows for a cascade of proactive measures: detailed patient counseling regarding potential complications like bladder/bowel injury and increased blood loss; scheduling the surgery during daytime hours with the most senior surgical team available; and ensuring the readiness of blood products and the potential intraoperative availability of a urologist or general surgeon. This preparedness can significantly enhance patient safety and optimize surgical outcomes [20].

However, our study is not without limitations. Firstly, being a single-center study, the generalizability of our findings to populations with different ethnicities, average BMIs, and surgical techniques may be limited. Secondly, while the operators were blinded, the assessment of striae and scars, though using standardized scales, has an inherent subjective component. Thirdly, the ultrasound sliding sign, while simple, is operator-dependent and requires training for consistent interpretation. Fourthly, our study did not account for other potential confounders of adhesion formation, such as the presence of subclinical endometritis, the technique of peritoneal closure in the previous surgery, or genetic polymorphisms related to fibrosis. Future multi-centric studies with larger sample sizes could incorporate these variables to develop a comprehensive predictive model or scoring system. Further research could also explore the combined predictive power of these markers, potentially deriving a composite score that offers even greater accuracy.

## Conclusions

In conclusion, our study provides compelling evidence that intra-abdominal adhesions in women undergoing repeat CS can be effectively predicted using a combination of easily accessible preoperative tools. The ultrasound sliding sign, the severity of abdominal striae graded by the Davey score, and the quality of the previous scar assessed by the VSS are all independently and significantly associated with intraoperative adhesion findings. The integration of this triad into routine preoperative assessment offers a pragmatic, non-invasive, and cost-effective strategy for risk stratification. By enabling obstetricians to anticipate and prepare for a potentially complicated surgery, this approach holds the promise of improving maternal safety and surgical outcomes in the face of the rising global CS epidemic.

## Appendices

**Table 4:** Questionnaire for the Study: Preoperative and Intraoperative Assessment of Adhesions

Section	Parameter	Question/observation	Response type/measurement
I. Demographic Details	Name/ID	Participant’s identification number	Open Field
	Age (years)	What is the participant’s age?	Numeric
	Gestational Age (weeks)	Gestational age at examination	Numeric
	BMI (kg/m²)	Calculated as weight (kg)/height² (m²)	Numeric
II. Obstetric History	Gravida/Parity	Record obstetric score (G_P_L_A)	Open Field
	Number of Previous Cesarean Sections	How many cesarean deliveries has the patient undergone?	1/2/≥3
	Interpregnancy Interval	Duration between last CS and current pregnancy (months)	Numeric
III. Clinical Evaluation	Presence of Striae	Are abdominal striae visible?	Yes/No
	Severity of Striae (Davey’s Score)	Score each abdominal quadrant (0–2); total (0–8)	None/Mild/Moderate/Severe
	Cesarean Scar Appearance	Describe scar morphology	Flat/Raised/Depressed
	Vancouver Scar Scale (VSS)	Record score for vascularity, pigmentation, pliability, height	≤4/>4
IV. Ultrasound Evaluation	Sliding Sign	Is uterine sliding visible on transabdominal ultrasound?	Positive/Negative
	Radiologist’s Observation	Interobserver consensus on sliding sign	Concordant/Discordant
V. Intraoperative Findings	Presence of Adhesions	Were adhesions observed intraoperatively?	Yes/No
	Adhesion Grade (Modified Tulandi and Lyell)	Intraoperative grading by the surgeon	Grade 0/1/2/3
	Adhesion Location	Sites involved	Uterus-Bladder/Omentum/Abdominal Wall
	Time for Peritoneal Entry (Minutes)	Duration from skin incision to uterine exposure	Numeric
VI. Outcome Variables	Intraoperative Complications	Any bladder/bowel injury, excessive bleeding, etc.	Yes/No (Specify)
	Postoperative Recovery	Duration of hospital stay (days)	Numeric
VII. Remarks	Surgeon’s Observation	Any special intraoperative note	Open Text
